# Repeat expansions in *AR*, *ATXN1*, *ATXN2* and *HTT* in Norwegian patients diagnosed with amyotrophic lateral sclerosis

**DOI:** 10.1093/braincomms/fcae087

**Published:** 2024-03-14

**Authors:** Camilla Novy, Øyvind L Busk, Ole-Bjørn Tysnes, Sigve S Landa, Tori N Aanjesen, Karl B Alstadhaug, Tale L Bjerknes, Ingrid K Bjørnå, Geir Bråthen, Elin Dahl, Natasha Demic, Maria Fahlström, Heidi Ø Flemmen, Erika Hallerstig, Ineke HogenEsch, Margitta T Kampman, Grethe Kleveland, Helene B Kvernmo, Unn Ljøstad, Angelina Maniaol, Aase Hagen Morsund, Ola Nakken, Cathrine G Olsen, Katrin Schlüter, May-Sissel Utvik, Ryaz Yaseen, Øystein L Holla, Trygve Holmøy, Helle Høyer

**Affiliations:** Department of Medical Genetics, Telemark Hospital Trust, 3710 Skien, Norway; Faculty of Medicine, Institute of Clinical Medicine, University of Oslo, 0316 Oslo, Norway; Department of Medical Genetics, Telemark Hospital Trust, 3710 Skien, Norway; Neuro-SysMed, Department of Neurology, Haukeland University Hospital, 5009 Bergen, Norway; Department of Medical Genetics, Telemark Hospital Trust, 3710 Skien, Norway; Department of Neurology, Akershus University Hospital, 1478 Lørenskog, Norway; Department of Neurology, Nordland Hospital Trust, 8005 Bodø, Norway; Neuro-SysMed, Department of Neurology, Haukeland University Hospital, 5009 Bergen, Norway; Institute of Clinical Medicine, University of Bergen, 5007 Bergen, Norway; Department of Neurology, Vestre Viken Hospital Trust, 3004 Drammen, Norway; Department of Neurology and Clinical Neurophysiology, St. Olavs Hospital, Trondheim University Hospital, 7030 Trondheim, Norway; Department of Neuromedicine and Movement Science, Norwegian University of Science and Technology, 7034 Trondheim, Norway; Department of Neurology, Telemark Hospital Trust, 3710 Skien, Norway; Department of Neurology, Vestfold Hospital Trust, 3103 Tønsberg, Norway; Department of Medical Genetics, Telemark Hospital Trust, 3710 Skien, Norway; Department of Neurology, Telemark Hospital Trust, 3710 Skien, Norway; Department of Neurology, Østfold Hospital Trust, 1714 Grålum, Norway; Department of Neurology, Fonna Hospital Trust, 5528 Haugesund, Norway; Department of Neurology, University Hospital of North Norway, 9019 Tromsø, Norway; Department of Neurology, Innlandet Hospital Trust, 2609 Lillehammer, Norway; Department of Neurology and Clinical Neurophysiology, St. Olavs Hospital, Trondheim University Hospital, 7030 Trondheim, Norway; Department of Neuromedicine and Movement Science, Norwegian University of Science and Technology, 7034 Trondheim, Norway; Institute of Clinical Medicine, University of Bergen, 5007 Bergen, Norway; Department of Neurology, Sørlandet Hospital Trust, 4615 Kristiansand, Norway; Department of Neurology, Oslo University Hospital, 0450 Oslo, Norway; Department of Neurology, Molde Hospital, 6412 Molde, Norway; Department of Neurology, Akershus University Hospital, 1478 Lørenskog, Norway; Department of Medical Genetics, Telemark Hospital Trust, 3710 Skien, Norway; Faculty of Medicine, Institute of Clinical Medicine, University of Oslo, 0316 Oslo, Norway; Department of Neurology, Stavanger University Hospital, 4019 Stavanger, Norway; Department of Neurology, Namsos Hospital Trust, 7803 Namsos, Norway; Department of Neurology, Oslo University Hospital, 0450 Oslo, Norway; Department of Medical Genetics, Telemark Hospital Trust, 3710 Skien, Norway; Faculty of Medicine, Institute of Clinical Medicine, University of Oslo, 0316 Oslo, Norway; Department of Neurology, Akershus University Hospital, 1478 Lørenskog, Norway; Department of Medical Genetics, Telemark Hospital Trust, 3710 Skien, Norway

**Keywords:** amyotrophic lateral sclerosis, genetic risk factor, Norway, population-based study

## Abstract

Genetic repeat expansions cause neuronal degeneration in amyotrophic lateral sclerosis as well as other neurodegenerative disorders such as spinocerebellar ataxia, Huntington’s disease and Kennedy’s disease. Repeat expansions in the same gene can cause multiple clinical phenotypes. We aimed to characterize repeat expansions in a Norwegian amyotrophic lateral sclerosis cohort. Norwegian amyotrophic lateral sclerosis patients (*n* = 414) and neurologically healthy controls adjusted for age and gender (*n* = 713) were investigated for repeat expansions in *AR*, *ATXN1*, *ATXN2* and *HTT* using short read exome sequencing and the ExpansionHunter software. Five amyotrophic lateral sclerosis patients (1.2%) and two controls (0.3%) carried ≥36 repeats in *HTT* (*P* = 0.032), and seven amyotrophic lateral sclerosis patients (1.7%) and three controls (0.4%) carried ≥29 repeats in *ATXN2* (*P* = 0.038). One male diagnosed with amyotrophic lateral sclerosis carried a pathogenic repeat expansion in *AR*, and his diagnosis was revised to Kennedy’s disease. In *ATXN1*, 50 amyotrophic lateral sclerosis patients (12.1%) and 96 controls (13.5%) carried ≥33 repeats (*P* = 0.753). None of the patients with repeat expansions in *ATXN2* or *HTT* had signs of Huntington’s disease or spinocerebellar ataxia type 2, based on a re-evaluation of medical records. The diagnosis of amyotrophic lateral sclerosis was confirmed in all patients, with the exception of one patient who had primary lateral sclerosis. Our findings indicate that repeat expansions in *HTT* and *ATXN2* are associated with increased likelihood of developing amyotrophic lateral sclerosis. Further studies are required to investigate the potential relationship between *HTT* repeat expansions and amyotrophic lateral sclerosis.

## Teaser

Novy *et al*. presents genetic repeat expansions in *AR*, *ATXN1*, *ATXN2* and *HTT* among 414 individuals diagnosed with amyotrophic lateral sclerosis (ALS) in a Norwegian cohort. Results indicate an increased risk of amyotrophic lateral sclerosis if carrying repeat expansions in *ATXN2* and *HTT*.

## Graphical Abstract

**Graphical Abstract:**
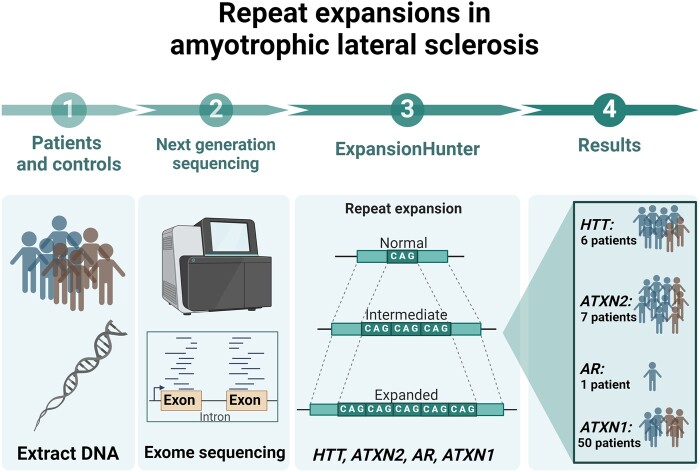

## Introduction

Amyotrophic lateral sclerosis is a rapidly progressive, fatal neurodegenerative disorder. Respiratory failure typically leads to death within two to four years of onset.^1^ Multiple genetic, metabolic and environmental risk factors have been identified.^2^ The most common genetic cause of amyotrophic lateral sclerosis in Europeans is a large hexanucleotide (GGGGCC) repeat expansion in *C9orf72*.^3^ The prevalence of this expansion in Norwegian patients (6.8%)^4^ is consistent with observed frequencies in other European amyotrophic lateral sclerosis cohorts (5–7%).^5^ Repeat expansions are also a cause of other neurodegenerative disorders, such as spinocerebellar ataxia, frontotemporal dementia, Huntington’s disease and Kennedy’s disease.^6,7^ Repeat expansion in a single gene can result in multiple clinical phenotypes; e.g. the *C9orf72* expansion may cause amyotrophic lateral sclerosis, frontotemporal dementia, or a combined phenotype.^8^ A *C9orf72* repeat expansion is the most common genetic cause of Huntington’s disease phenocopies.^9^

Trinucleotide (CAG) repeat expansions in *HTT* cause Huntington’s disease and have also been reported in individuals with amyotrophic lateral sclerosis,^7,10,11^ suggesting an overlap in molecular pathology.^10,12,13^  *HTT* alleles with ≥40 repeats are disease-causing, whereas alleles with 36–39 repeats exhibit reduced penetrance. Huntington’s disease usually presents in the fourth decade, and longer repeat sizes are associated with earlier onset and higher penetrance.^14^

Repeat expansions in *ATXN1* and *ATXN2* cause spinocerebellar ataxia types 1 and 2, respectively. In 2010, intermediate-length repeat expansions in *ATXN2* were reported to be a risk factor for amyotrophic lateral sclerosis,^15^ and in 2012, the same was reported for *ATXN1*.^16^ Subsequently, intermediate expansions in either *ATXN2* (29–33 CAG repeats), or *ATXN1* (≥33 CAG repeats), were shown to increase the risk of developing amyotrophic lateral sclerosis.^17,18^

Repeat expansions (CAG) in *AR* cause Kennedy’s disease, an X-linked recessive motor neuron disease, which affects hemizygous males. In two previous studies, 2% and 3.7% of patients diagnosed with amyotrophic lateral sclerosis carried a pathogenic repeat expansion in *AR*.^7,19^

Large and intermediate repeat expansions can be difficult to detect on exome short-read sequencing data,^20^ a standard diagnostic method. Software like ExpansionHunter^21,22^ has made it possible to detect pathogenic repeat expansions in short-read sequencing data. Most studies have used ExpansionHunter on whole-genome sequencing data,^7,23^ but few studies have been able to detect repeat expansions for specific loci in exome data.^24,25^

Using an exome sequencing approach with ExpansionHunter,^21,22^ we investigated a Norwegian amyotrophic lateral sclerosis cohort for repeat expansions in *AR*, *ATXN1*, *ATXN2* and *HTT*.

## Materials and methods

### Patients and controls

Our amyotrophic lateral sclerosis cohort consisted of 414 individuals diagnosed clinically with amyotrophic lateral sclerosis by a neurologist, with a median age of 65.5 years (range 27–87 years) (Supplementary Fig. 1). Participants were included from 17 study sites throughout Norway between August 2019 and August 2022. The neurologists completed a questionnaire for each individual, which focused on diagnostic certainty and clinical characteristics, including signs of upper and lower motor neuron involvement, cognitive function, results of neurophysiological examination and whether El Escorial diagnostic criteria^26^ were met. Patients provided information regarding family history of amyotrophic lateral sclerosis or other neurodegenerative disorders in first-degree, second-degree or other relatives. A family history of amyotrophic lateral sclerosis was reported in 50 affected individuals (12.1%): 38 with first-degree relatives and 12 with second-degree relatives. For patients with pathogenic repeat expansions in *ATXN2*, *HTT* and *AR*, clinical data were retrieved from medical records and re-evaluated independently by two experienced neurologists. Anonymized neurologically normal controls (*n* = 713) with a median age of 47.0 years (Supplementary Fig. 1) were obtained from the diagnostic laboratory, Department of Medical Genetics, Telemark Hospital Trust. Controls were selected from individuals not being investigated for a neurological disorder (Supplementary Table 1) who were 25 years of age or older (Supplementary Fig. 2).

Written informed consent was obtained from all affected individuals. The study was approved by the Norwegian Regional Committees for Medical and Health Research Ethics (2018/1916), the Norwegian Centre for Research Data and the Data Protection Officers at participating hospitals. De-identified data from individuals with ataxia were made available after approval by the Norwegian Centre for Research Data and the data protection officer at Telemark Hospital Trust.

### Exome sequencing

Whole-exome sequencing was performed on an Illumina NextSeq 500 instrument using the Human Core Exome EF Multiplex Kit or the Exome 2.0 Kit (Twist Bioscience) following standard procedures. Targeted bases consisted of all coding exons and flanking intron sequences. Reads were mapped to the reference genome (GRCh37/hg19) using the Burrows–Wheeler Alignment tool.^27^ In the majority of the samples, 95% of all targeted bases had a coverage > 20×. Samples with a coverage < 90% at 20× were reanalysed.

### ExpansionHunter

We validated ExpansionHunter software (v.5.0.0)^21,22^ results using variant data from ataxia patients (*n* = 88) who had previously been analysed with standard PCR-based fragment analyses of *ATXN1* and *ATXN2* with an error margin of plus or minus one repeat. Thereafter, ExpansionHunter software was used to detect CAG repeat expansions in *AR*, *ATXN1*, *ATXN2* and *HTT* with default parameters as specified in Supplementary Table 2. Reference ranges for normal, intermediate and pathogenic repeat sizes in *AR*, *ATXN1*, *ATXN2* and *HTT* were based on data from updated summaries^28-32^ (Supplementary Table 3). Repeat expansions were classified as expanded if above the cut-off for a normal repeat length. Visual inspection was performed for affected individuals with Integrative Genome Viewer (IGV)^33^ software for repeat expansions that ExpansionHunter was not able to detect due to low coverage. Unfortunately, it was not possible to identify and visually inspect controls that had been anonymized prior to the analysis. The coverage of repeat expansions in *AR*, *ATXN1*, *ATXN2* and *HTT* in cases and controls is shown in the Supplementary Fig. 3.

### Genetic analysis of known motor neuron disease genes

Amyotrophic lateral sclerosis patients carrying repeat expansions in *AR*, *ATXN2* and *HTT* were analysed using NGS for variants in 70 genes known to be associated with amyotrophic lateral sclerosis and other motor neuron disorders (Supplementary Table 4).

### Fragment length analyses of *AR*, *HTT*, *ATXN1*, *ATXN2* and *C9orf72*

We used fragment length analysis to confirm allele size in borderline normal/intermediate, intermediate and expanded ranges in amyotrophic lateral sclerosis patients. PCR-based fragment length analysis was performed according to standard procedures using PCR with fluorescent-labelled primers and electrophoretic length separation of PCR products (Supplementary Table 5). Repeat primed PCR (RP-PCR) was performed using standard procedures to confirm or rule out homoallelism, and to detect interrupted repeats in *ATXN1* and *ATNX2*. All individuals in the amyotrophic lateral sclerosis cohort had previously been investigated for the intronic *C9orf72* repeat expansion, not detectable by exome sequencing, using the Asuragen Amplide PCR/CE *C9orf72* kit.

### Statistical analyses

To assess the detection performance of repeat expansions in NGS data by ExpansionHunter compared to standard PCR-based fragment analyses, sensitivity and specificity were calculated by the proportion of expanded and non-expanded alleles among PCR-confirmed alleles given an error margin of plus or minus one repeat. A more detailed description of the statistical formula is provided in the Supplementary material (p 4). Logistic regression analyses (two-tailed) were applied to detect statistically significant differences in the prevalence of repeats in *ATXN*1, *ATXN2* and *HTT* (*P* < 0.05) among amyotrophic lateral sclerosis patients and controls, adjusting for age and gender. Logistic regression analysis was not performed for *AR* due to the low number of expanded alleles. Statistical analyses were performed using Stata software (v.17.0).^34^

## Results

**Figure 1:**
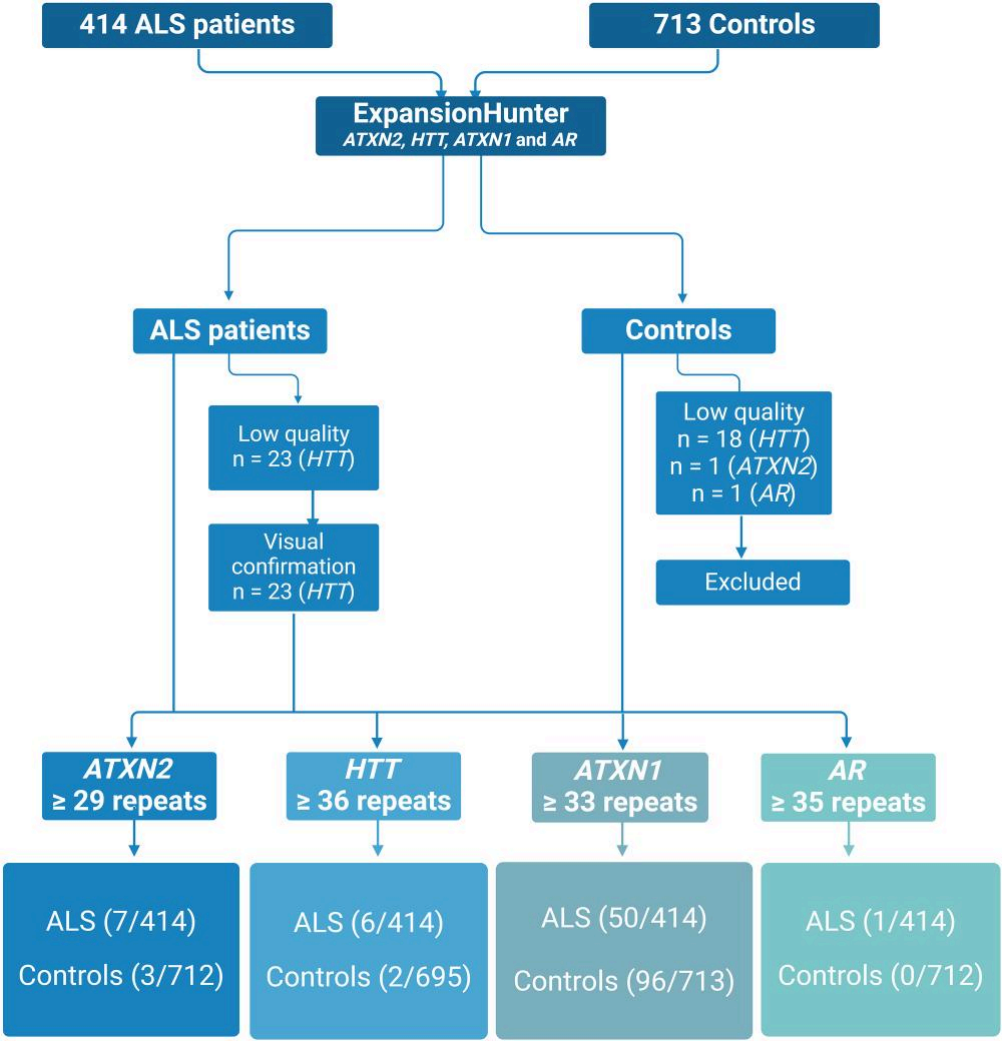
**Repeat expansions detection workflow.** ExpansionHunter software is performed on exome sequencing data. Low coverage samples can be investigated for visual confirmation. Expanded repeats in *HTT* and *ATXN2* were found in 13 amyotrophic lateral sclerosis patients. Created with BioRender.com.

Repeat expansion analyses were performed for 414 amyotrophic lateral sclerosis patients and 713 neurologically normal controls; pathogenic and intermediate-length repeat expansions in *AR*, *ATXN2* and *HTT* were observed in 3.1% of the amyotrophic lateral sclerosis patients (Fig. 1). This included repeat expansions associated with full penetrance in *AR* (*n* = 1) and *HTT* (*n* = 1), repeat expansions associated with reduced penetrance in *ATXN2* (*n* = 2) and *HTT* (*n* = 5) and intermediate repeat expansions in *ATXN2* (*n* = 5). We identified intermediate repeat expansions in *ATXN1*, but there was no difference between cases (*n* = 50) and controls (*n* = 96).

### HTT

**Figure 2:**
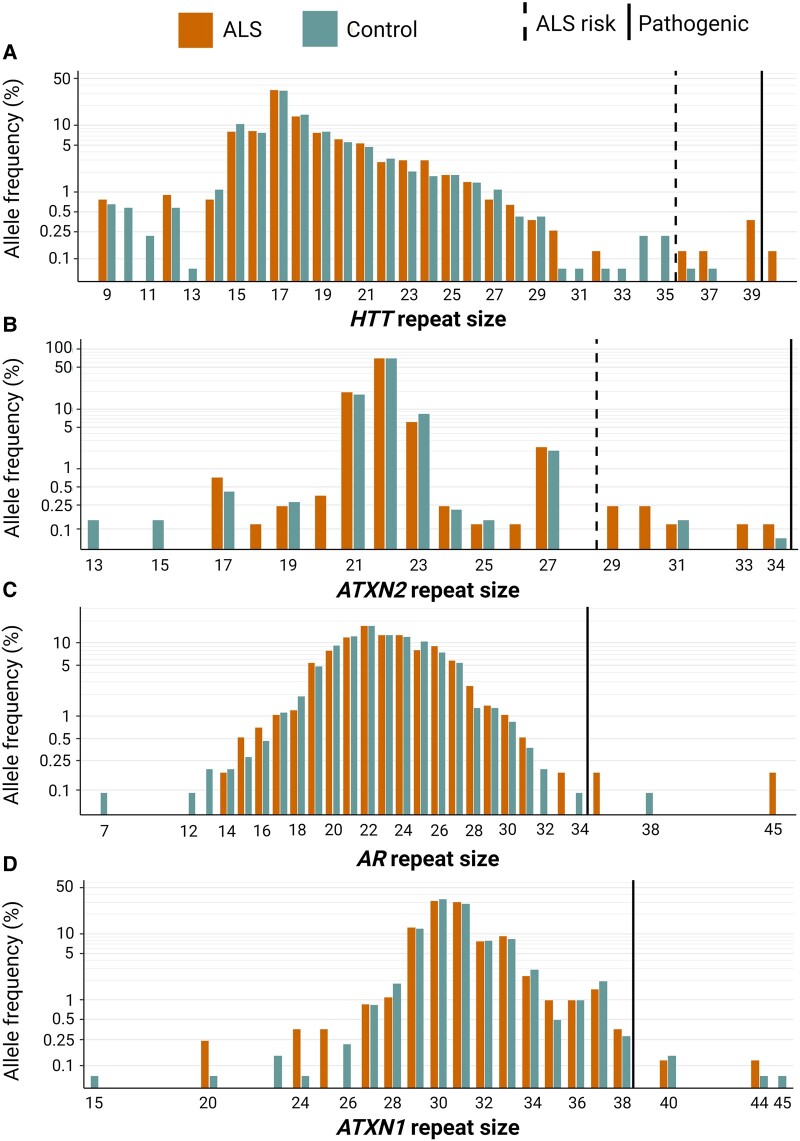
**Distribution of allele frequency and allele sizes in amyotrophic lateral sclerosis patients and controls.** (**A**) *HTT* repeat expansions in 391 amyotrophic lateral sclerosis patients and 695 controls. (**B**) *ATXN2* repeat expansions in 414 amyotrophic lateral sclerosis patients and 712 controls. (**C**) *AR* repeat expansions in 414 amyotrophic lateral sclerosis patients and 712 controls and (**D**) *ATXN1* repeat expansions in 414 amyotrophic lateral sclerosis patients and 713 controls. The number of amyotrophic lateral sclerosis patients and controls differs since samples with low coverage for a specific repeat were removed from the analysis. The dashed black line represents the thresholds of which the number of repeats is considered to be associated with amyotrophic lateral sclerosis risk, and the black line represents the thresholds of which the number of repeats is considered to be pathogenic for each locus (Supplementary Table 3). Created with BioRender.com.

We identified six amyotrophic lateral sclerosis patients (1.5%) carrying ≥36 repeats (36, 37, 39, 39, 39 and 40) in *HTT* (Fig. 1). In the control group (*n* = 695), two individuals carried repeat expansions (0.3%) in the reduced penetrance range (36 and 37 repeats). One amyotrophic lateral sclerosis patient with 36 repeats also carried a high-penetrant pathogenic variant in an established amyotrophic lateral sclerosis gene that corresponded well with the clinical amyotrophic lateral sclerosis symptoms. This individual was excluded from further statistical analyses. Repeat expansions in *HTT* (≥36 repeats) were associated with a higher risk of amyotrophic lateral sclerosis (OR 6.99, 95% CI 1.2–41.5, *P* = 0.03) (Table 1). Repeat sizes ranged from nine to 40 in the amyotrophic lateral sclerosis group and from nine to 37 in the control group (Fig. 2A). The ExpansionHunter software did not validate the data quality in 18 controls (2.5%) because of insufficient coverage, and these individuals were not included in the analyses. Amyotrophic lateral sclerosis samples that did not pass the ExpansionHunter software quality control (*n* = 23, 5.6%) were visually inspected and manually confirmed. The median age at onset for amyotrophic lateral sclerosis patients with *HTT* repeat expansions was 58 years, and in non-carriers 66 years.

**Table 1:** Amyotrophic lateral sclerosis risk associated with expansions of *ATXN1*, *ATXN2* and *HTT*

Repeat size	Amyotrophic lateral sclerosis		Controls		Logistic regression	
	*n*	%	*n*	%	OR (95% CI)	*P*-value
*ATXN1*						
≥33 repeats	50	12.08	96	13.46	0.94 (0.63–1.41)	0.753
<33 repeats	364	87.92	617	86.54		
*ATXN2*						
≥29 repeats	7	1.69	3	0.42	4.81 (1.09–21.18)	**0**.**038**
<29 repeats	407	98.31	709	99.58		
*HTT*						
≥36 repeats	5	1.21	2	0.29	6.99 (1.18–41.52)	**0**.**032**
<36 repeats	408	98.79	693	99.71		

Bold values indicate significant *P*-values.

None of the patients with *HTT* repeat expansions had psychiatric symptoms or involuntary movements suggestive of Huntington’s disease, and the amyotrophic lateral sclerosis diagnosis was confirmed after re-evaluation of medical records. In the five patients with a repeat expansion in *HTT*, there were one female and four males with an age of onset between the fourth and eighth decades of life. All patients had a spinal onset and one had cognitive impairment. All five patients with *HTT* repeat expansions had upper and lower motor neuron signs and fulfilled the El Escorial criteria.^26^ None of the patients reported a family history of amyotrophic lateral sclerosis but two patients carried pathogenic variants known to increase the risk of amyotrophic lateral sclerosis. Four of the patients with repeat expansions in *HTT*, were alive after at least 76 months after onset.

### ATXN2

We identified seven amyotrophic lateral sclerosis patients (1.7%) carrying ≥29 repeats (29, 29, 30, 30, 31, 33 and 34) in *ATXN2* (Fig. 1) compared to three (0.4%) in the control group (31, 31 and 34 repeats) (OR 4.8, 95% CI 1.1–21.2, *P* = 0.04) (Table 1). In these seven patients, there were three females and four males with an age of onset between the fourth and eighth decades of life. All patients had a spinal onset, except one with a bulbar onset. None had cognitive impairment. None of the patients reported a family history of amyotrophic lateral sclerosis. With the exception of one patient who had a primary lateral sclerosis phenotype, all patients fulfilled the El Escorial criteria.^26^ One control did not pass the ExpansionHunter software quality control and was therefore not included in the analysis. In amyotrophic lateral sclerosis patients, repeat sizes ranged from 17 to 34, and in the control group from 13 to 34 (Fig. 2B). Five amyotrophic lateral sclerosis patients carried repeat expansions in the amyotrophic lateral sclerosis risk range of *ATXN2* (29–31), including one who had an uninterrupted repeat expansion. Two patients carried expanded *ATXN2* alleles in the range associated with reduced penetrance for spinocerebellar ataxia type 2 (33 and 34 repeats). One of these carried an interrupted repeat expansion, and the amyotrophic lateral sclerosis diagnosis was confirmed after a re-evaluation of medical records. The other patient, who had primary lateral sclerosis, had an uninterrupted repeat expansion. These two patients had no clinical symptoms typical of spinocerebellar ataxia type 2, e.g. ataxia, incoordination, or ophthalmoplegia.

### AR

A pathogenic repeat expansion in *AR* was observed in one male patient (0.2%) diagnosed with amyotrophic lateral sclerosis. He had axonal motor and sensory polyneuropathy and gynaecomastia characteristic of Kennedy’s disease, and no other upper motor signs than brisk tendon reflexes. The diagnosis was therefore revised accordingly. No pathogenic repeat expansion in *AR* was observed in the control group. One control sample did not pass the ExpansionHunter software quality control and was not included in the analysis (Fig. 1). One female amyotrophic lateral sclerosis patient and one female control carried heterozygous repeat expansions (35 repeats and 38 repeats) (Fig. 2C).

### ATXN1

Repeat expansions in *ATXN1* (≥33 repeats) were carried by 50 amyotrophic lateral sclerosis patients (Fig. 1). There was no association between the risk of developing amyotrophic lateral sclerosis and having ≥33 repeats in *ATXN1* (Table 1). We identified two amyotrophic lateral sclerosis patients who carried repeat expansions in the expanded range (40 and 44 repeats). Both expansions were interrupted by a CAT repeat and therefore not considered pathogenic for spinocerebellar ataxia type 1.^35,36^ Repeat sizes ranged from 20 to 44 in the amyotrophic lateral sclerosis group and from 15 to 45 in the control group (Fig. 2D).

### Validation of ExpansionHunter for *ATXN1* and *ATXN2*

Results obtained using ExpansionHunter software were validated with PCR in 88 ataxia patients. The number of repeats detected in *ATXN1* was identical for 17/176 alleles, 158/176 alleles showed plus one repeat with ExpansionHunter compared to PCR analysis and 1/176 showed plus two repeats with ExpansionHunter compared to PCR analysis. The number of repeats detected in *ATXN2* was identical for 156/176 alleles, and 20/176 alleles differed with plus one repeat using ExpansionHunter software compared to PCR analysis. Given an error margin of plus or minus one repeat, sensitivity for ExpansionHunter was 100.0% (95% CI 39.8–100.0%) for *ATXN1* and *ATXN2*, and specificity was 99.4% (95% CI 96.8–99.9%) for *ATXN1* and 100.0% (95% CI 97.9–100.0%) for *ATXN2* (Supplementary Table 6). Since the majority of alleles (158/176) showed plus one repeat for *ATXN1* with ExpansionHunter compared to PCR analysis, all amyotrophic lateral sclerosis patients and controls were adjusted accordingly with minus one repeat for *ATXN1* in the logistic regression analysis.

## Discussion

In this cohort study, *HTT* and *ATXN2* repeat expansions were associated with an increased risk of developing amyotrophic lateral sclerosis.

The association between amyotrophic lateral sclerosis and *HTT* repeat expansions has been reported in previous cohort and case studies.^12,37^ However, the prevalence of repeat expansions in *HTT* is higher in our amyotrophic lateral sclerosis cohort (1.5%) than previously reported (0.2%).^11,38^ This could be because of the misdiagnosis of Huntington’s disease as amyotrophic lateral sclerosis. However, experienced neurologists monitored all amyotrophic lateral sclerosis cases every three months, and two independent amyotrophic lateral sclerosis experts reviewed the medical files. The re-evaluation of medical records did not reveal psychiatric symptoms or involuntary movements suggestive of Huntington’s disease. Patients, and in many instances their close relatives, were questioned about family members with other neurological diseases. Thus, while it is unlikely, the chance remains that a positive family history of Huntington’s disease could have been missed. The absence of a Huntington’s disease phenotype in patients with expanded *HTT* alleles might reflect the older age of onset observed in Huntington’s disease patients with short repeat expansions (36–39) in *HTT*.^39^ Two individuals with repeat expansions in *HTT* also carried pathogenic variants known to increase the risk of amyotrophic lateral sclerosis. It can be speculated that the combination of these variants and a repeat expansion in *HTT* had an impact on their disease phenotype. One case carried 36 repeats in *HTT* that is in the lower penetrance range. Since this individual also carried a pathogenic variant in an established amyotrophic lateral sclerosis gene and had typical corresponding clinical manifestations, we considered it unlikely that the repeat expansion in *HTT* had an impact on the phenotype. This individual was therefore removed from the statistical analysis.

In our control group, we identified two individuals carrying 36 and 37 repeats in *HTT*. Repeat expansion sizes up to 38 repeats have been identified in other control groups.^11^ Kay *et al.*^40^ found that repeat sizes of 36–38 occur in 0.3% of the general population. This is compatible with the prevalence in our control group (0.3%). Uninterrupted trinucleotide repeats in the reduced penetrance range can modify the age of onset.^41^ Unfortunately, the method we used was not able to detect such interruptions.

Further studies are needed to evaluate the possible effect of *HTT* repeat expansions on the clinical features of amyotrophic lateral sclerosis. Ongoing clinical trials targeting *HTT* repeat expansions in Huntington’s disease (e.g. Clinical Trial NCT05534139) may have the potential to identify future therapeutics relevant to amyotrophic lateral sclerosis patients carrying *HTT* repeat expansions.

For *ATXN2*, our findings are similar to those previously reported in other cohorts (e.g. British and Dutch), where Sproviero *et al*.^42^ confirmed increased amyotrophic lateral sclerosis risk for individuals carrying 29–33 repeats and an exponential risk increase of amyotrophic lateral sclerosis with repeat size. Two amyotrophic lateral sclerosis patients in our cohort carried ≥33 repeats in *ATXN2*, a cause of spinocerebellar ataxia type 2. One of these patients has primary lateral sclerosis, whereas the other patient had both upper and lower motor neuron involvement and died from amyotrophic lateral sclerosis. Our findings are relevant for ongoing clinical trials that recruit amyotrophic lateral sclerosis patients with repeat expansions in *ATXN2* (Clinical Trial NCT04494256), where a drug that reduces the level of ataxin-2 protein is under investigation.

Only one patient (0.24%) in the amyotrophic lateral sclerosis cohort carried a hemizygous repeat expansion in *AR*, leading to a revised diagnosis of Kennedy’s disease. We expected to identify a higher number of such patients based on Parboosingh *et al*.^19^ findings showing 2% misdiagnoses in amyotrophic lateral sclerosis patients and Ibañez *et al*.,^7^ who found that 3.7% of patients diagnosed with amyotrophic lateral sclerosis carried pathogenic repeat expansions in *AR*. This discrepancy could reflect differences in patient selection, diagnostic practice, or prevalence of Kennedy’s disease.

We did not find any association between repeat expansions in the *ATXN1* gene and the risk of amyotrophic lateral sclerosis. Our findings diverge from other studies,^17,18^ and the reason for this disparity is unclear. However, we observed a high frequency of ≥33 repeats in the control group (13.5%) compared to other studies (8.8% and 5.5%)^17,18^ that could indicate a higher prevalence of such repeats in the Norwegian population.

Why repeat expansions in various genes are associated with increased amyotrophic lateral sclerosis risk is not known. However, protein products of *ATXN2* interact with the TAR DNA binding protein-43 (TDP-43) resulting in TDP-43 mislocalization and aggregate formation, a hallmark of amyotrophic lateral sclerosis.^15^ Tazelaar *et al.*^18^ discovered that overexpression of ataxin-1 disturbs the nucleocytoplasmic transport of TDP-43. Coudert *et al*.^43^ showed that aggregation and phosphorylation of TDP-43 were induced in cells with a *HTT* repeat expansion and that mutant Huntingtin (HTT) co-localized with TDP-43, supporting that repeat expansions in *HTT* are a potential genetic risk factor for amyotrophic lateral sclerosis.

In this study, we were able to detect repeat expansions with the ExpansionHunter software on exome sequencing data, circumventing the need for a supplemental PCR-based method. Limitations with exome sequencing data are the lack of coverage in areas situated outside the coding region and the short reads,^25^ excluding large intronic expansions such as *C9orf72*. Moreover, the variability in target coverage can be a challenge with exome sequencing, requiring a visual inspection of repeat expansions.^24^ We experienced that ExpansionHunter was vulnerable to low sequence coverage, especially for *HTT*. The validation of ExpansionHunter for *ATXN1* and *ATXN2* showed that some alleles were shifted by one or two repeats. ExpansionHunter was not validated for *AR* and *HTT*, thus we cannot exclude a similar shift. As performed in this study, we recommend additional PCR testing when allele sizes are plus or minus two repeats from a cut-off value and visual inspection when samples do not pass the ExpansionHunter software quality control due to low coverage.

This study’s main limitation is the small sample size of the amyotrophic lateral sclerosis cohort. In addition, our control group consisted of neurologically healthy individuals but included individuals diagnosed with other disorders and is not representative of an unselected neurologically normal population. Further, since the controls had been anonymized, it was not possible to visually inspect or verify the results by PCR. The vast majority of Norwegian amyotrophic lateral sclerosis patients are followed regularly in amyotrophic lateral sclerosis clinics at their local hospitals. We were therefore able to include a large proportion of all amyotrophic lateral sclerosis cases. It is, however, likely that this approach will be selected for patients with slow progression and long survival.

In conclusion, our findings suggest that repeat expansions in *HTT* could increase the risk of developing amyotrophic lateral sclerosis. Further studies are required to investigate this possibility and to understand the potential relationship between *HTT* repeat expansions and amyotrophic lateral sclerosis. In line with previous studies, we observed a higher risk of developing amyotrophic lateral sclerosis in patients carrying repeat expansions in *ATXN2*, but we did not find an association between amyotrophic lateral sclerosis risk and repeat expansions in *ATXN1*. We observed a lower frequency of repeat expansions in *AR* compared to previous studies on amyotrophic lateral sclerosis patients.

## Supplementary Material

fcae087_Supplementary_Data

## Data Availability

The data that supports the findings of this study are available from the corresponding author, upon reasonable request. ExpansionHunter is freely available at https://github.com/Illumina/ExpansionHunter, and the scripts used in this study are available in the Supplementary material.
