# Supplementary material for: Repeat expansions in AR, ATXN1, ATXN2 and HTT in Norwegian patients diagnosed with amyotrophic lateral sclerosis
Source: Brain Commun. 2024 Mar 14;6(2):fcae087. doi: 10.1093/braincomms/fcae087 (PMC10998343; doi:10.1093/braincomms/fcae087)
Supplement: fcae087_Supplementary_Data [file fcae087_supplementary_data.docx]

# Supplementary material

## Controls

**Supplementary Table 1 Neurologically normal controls (n = 713) from all parts of Norway were obtained from the diagnostic laboratory, Department of medical genetics, Telemark Hospital Trust. Inclusion criteria was age above 25 years (range 25 to 86 years). This cohort included patients investigated for various diseases, excluding neurological diseases.**

| **Disease** | ***n*** |
| --- | --- |
| Amylogenesis imperfecta | 1 |
| Arthrosis | 1 |
| Bleeding disorders | 57 |
| Bone marrow disorders | 41 |
| Custom analysis^a^ | 91 |
| Cystic fibrosis | 15 |
| Developmental disorders | 252 |
| Erythrocytosis | 11 |
| Immune deficiency | 27 |
| Lung fibrosis | 143 |
| Macrocephaly | 2 |
| Neurofibromatosis type 1 | 1 |
| Osteogenesis imperfecta | 12 |
| Prostate cancer | 43 |
| Skeletal disease | 5 |
| Schwannomatosis | 3 |
| Thoracic aortic aneurysm and dissection | 8 |

^a^Diverse indications excluding motor neuron disease

## Comparison of age between cases and controls


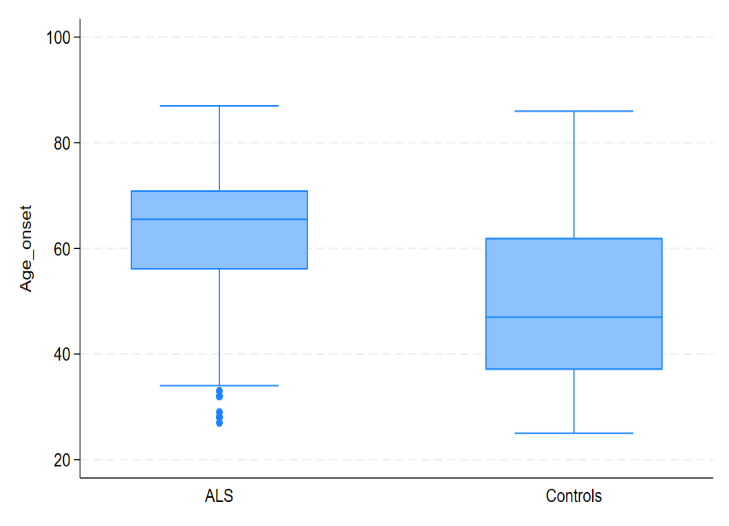


**Supplementary Figure 1 Boxplot of the age distribution for ALS patients and controls.** ALS patients had a median age of 65.5 years and controls had a median age of 47.0 years.


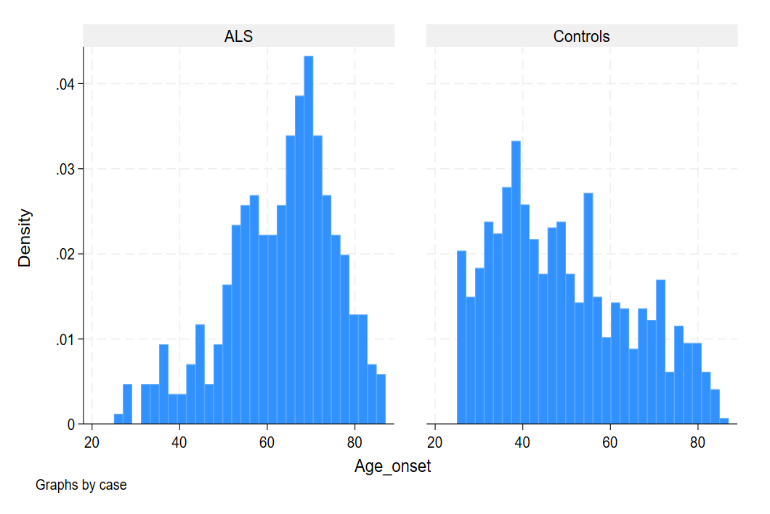


**Supplementary Figure 2 Histogram of the age distribution for both ALS cases and controls.** The age of ALS cases ranged from 27 to 87 years and controls from 25 to 86 years.

## ExpansionHunter catalog

Here is an example for how the catalog design looks for each locus (e.g. ATXN1).

"VariantType": "Repeat",

"LocusId": "ATXN1",

"LocusStructure": "(TGC)*",

"ReferenceRegion": "6:16327864-16327954"

**Supplementary Table 2 Reference region for each locus in the catalog**

| **Locus ID** | **Locus Structure** | **Reference Region (GRCh37)** |
| --- | --- | --- |
| AR | (GCA)* | chrX: 66765158-66765227 |
| ATXN1 | (TGC)* | chr6: 16327864-16327954 |
| ATXN2 | (GCT)* | chr12: 112036753-112036822 |
| HTT | (CAG)* | chr4: 3076603-3076660 |

## Repeat expansion loci and diseases

Kennedy’s disease (AR; OMIM # 313200, NM_000044.6),^1^ Spinocerebellar ataxia type 1 (ATXN1; OMIM #164400, NM_001128164.2)^2^, Spinocerebellar ataxia type 2 (ATXN2; OMIM #183090, NM_002973.3)^3^, and Huntington’s disease (HTT; OMIM # 143100, NM_001388492.1 )^4^.

## Repeat expansion size overview

**Supplementary Table 3 Reference ranges for normal, intermediate and pathogenic repeat sizes.**

| **Gene** | **Disease** | **Normal** | **Intermediate** | **Pathogenic** | |
| --- | --- | --- | --- | --- | --- |
|  |  |  |  | **Reduced penetrance** | **Full-Penetrance** |
| *AR^5^* | Kennedy's disease | 10-34 | - | 35-37 | ≥38 |
| *ATXN1^6^* | Spinocerebellar ataxia type 1 | 6-32 | 33-35 | 36-44^a^ | ≥39 |
| *ATXN2^6^* | Spinocerebellar ataxia type 2 | ≤26 | 27-31 | 32-34 | ≥35 |
| *C9orf72^7,8^* | Frontotemporal dementia or ALS | 2-24 | 25-60 | 25-60^b^ | >60 |
| *HTT^9^* | Huntington's disease | ≤26 | 27-35 | 36-39 | ≥40 |

^a^Without CAT interruptions

^b^Unknown significance of penetrance

## Repeat expansion coverage


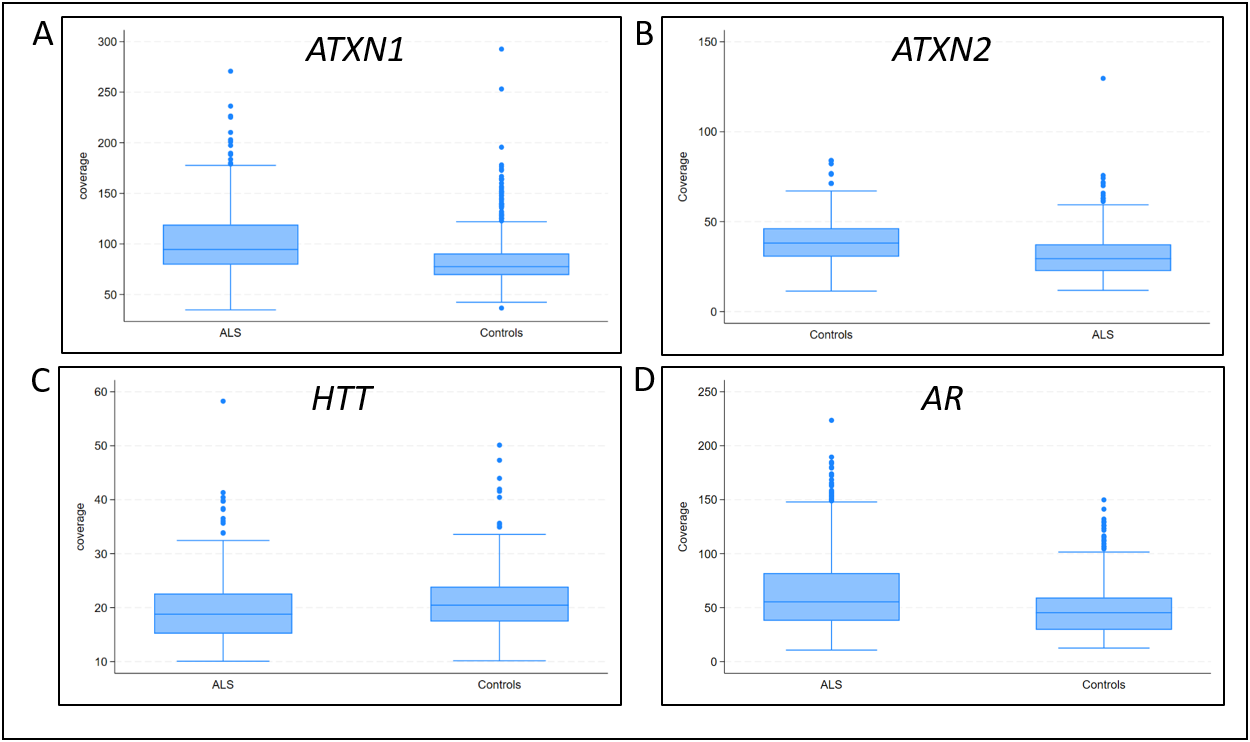


**Supplementary Figure 3 Boxplot of exome sequencing coverage in the repeated loci of AR, ATXN1, ATXN2, and HTT among ALS cases and controls.** The minimum coverage to pass ExpansionHunter is 10**.** (**A**) Loci coverage for the repeat expansion in ATXN1. (**B**) Loci coverage for the repeat expansion in ATXN2. (**C**) Loci coverage for the repeat expansion in HTT. (**D**) Loci coverage for the repeat expansion in AR.

## Gene panel

**Supplementary Table 4 Genes analysed for ALS patients carrying repeat expansions.**

| **# Genes** | **Gene** | **# Genes** | **Gene** | **# Genes** | **Gene** | **# Genes** | **Gene** |
| --- | --- | --- | --- | --- | --- | --- | --- |
| 1 | *AAAS* | 19 | *DYNC1H1* | 37 | *MATR3* | 55 | *SORD* |
| 2 | *AARS1* | 20 | *ERBB3* | 38 | *NEFH* | 56 | *SPG11* |
| 3 | *ALS2* | 21 | *ERBB4* | 39 | *OPTN* | 57 | *SPTLC1* |
| 4 | *ANG* | 22 | *EXOSC3* | 40 | *PFN1* | 58 | *SQSTM1* |
| 5 | *ANXA11* | 23 | *EXOSC8* | 41 | *PIP5K1C* | 59 | *SS18L1* |
| 6 | *ASAH1* | 24 | *FBXO38* | 42 | *PLEKHG5* | 60 | *TARDBP* |
| 7 | *ASCC1* | 25 | *FIG4* | 43 | *POLG* | 61 | *TBK1* |
| 8 | *ATP7A* | 26 | *FUS* | 44 | *RBM7* | 62 | *TRIP4* |
| 9 | *ATXN1* | 27 | *GARS1* | 45 | *REEP1* | 63 | *TRPV4* |
| 10 | *ATXN2* | 28 | *GLE1* | 46 | *SETX* | 64 | *TUBA4A* |
| 11 | *BICD2* | 29 | *GLT8D1* | 47 | *SIGMAR1* | 65 | *UBA1* |
| 12 | *BSCL2* | 30 | *HEXB* | 48 | *SLC52A1* | 66 | *UBQLN2* |
| 13 | *CCNF* | 31 | *HNRNPA1* | 49 | *SLC52A2* | 67 | *VAPB* |
| 14 | *CHCHD10* | 32 | *HSPB1* | 50 | *SLC52A3* | 68 | *VCP* |
| 15 | *CHMP2B* | 33 | *HSPB3* | 51 | *SLC5A7* | 69 | *VRK1* |
| 16 | *DAO* | 34 | *HSPB8* | 52 | *SMN1* | 70 | *WARS1* |
| 17 | *DCTN1* | 35 | *IGHMBP2* | 53 | *SMN2* |  |  |
| 18 | *DNAJB2* | 36 | *KIF5A* | 54 | *SOD1* |  |  |

## PCR-analysis primers

**Supplementary Table 5 Primer sequences used in PCR analyses**

| **Gene** | **Forward primer** | **Fluorescent tag** | **Reverse primer** |
| --- | --- | --- | --- |
| *AR* | GCCGCGAGCGCAGCACCTCC | FAM | GGGAGAACCATCCTCACCCT |
| *ATXN1* | AACTGGAAATGTGGACGTAC | NED | CAACATGGGCAGTCTGAG |
| *ATXN1** | GGGCCCCTCACCATGTCG | VIC | TACGCATCCCAGTTTGAGACG |
| *ATXN2* | CGTGCGAGCCGGTGTATGGG | PET | GGCGACGCTAGAAGGCCGCT |
| *ATXN2** | AACTGGAAATGTGGACGTAC | NED | TACGCATCCCAGTTTGAGACG |
| *HTT* | CCTTCGAGTCCCTCAAGTCCTTC | FAM | CGGCGGTGGCGGCTGTTG |

*RP-PCR

## Statistical analysis

### Validation of ExpansionHunter

Statistic formulas was retrieved from MedCalc Software Ltd. Diagnostic test evaluation calculator (https://www.medcalc.org/calc/diagnostic_test.php) (Version 20.211; accessed January 4, 2023), to calculate performance of repeat expansion detection by ExpansionHunter.

**Supplementary Table 6 Calculated sensitivity and specificity per allele, for detection performance of ExpansionHunter (given acceptance of +/- one repeat), validated by PCR analysis**.

| **Loci** | **TN** | **FN** | **TP** | **FP** | **Sensitivity** | **95% CI** | **Specificity** | **95% CI** | **PPV** | **NPV** |
| --- | --- | --- | --- | --- | --- | --- | --- | --- | --- | --- |
| ATXN1 | 173 | 0 | 2 | 1 | 100% | 15.81-100.00 | 99.43% | 96.84-99.99 | 0.26% | 100% |
| ATXN2 | 174 | 0 | 2 | 0 | 100% | 15.81-100 | 100% | 97.90-100.00 | 100% | 100% |
| **Total** | **347** | **0** | **4** | **1** | **100%** | **39.76-100.00** | **99.71%** | **98.41-99.99** | **0.52%** | **100%** |

TP = True positive, FP = False positive, TN = True negative, FN = False negative, PPV = Positive predicted value, NPV = Negative predicted value.

Thresholds for positive is the thresholds for pathogenic repeat sizes (ATXN1; >=39, ATXN2; >=33). The prevalence of Spinocerebellar ataxia type 1 (ATXN1) and Spinocerebellar ataxia type 2 (ATXN2) is 0.0015% (1-2:100 000).^10^

## References

1. La Spada AR, Wilson EM, Lubahn DB, Harding AE, Fischbeck KH. Androgen receptor gene mutations in X-linked spinal and bulbar muscular atrophy. *Nature*. Jul 4 1991;352(6330):77-9. doi:10.1038/352077a0

2. Orr HT, Chung MY, Banfi S, et al. Expansion of an unstable trinucleotide CAG repeat in spinocerebellar ataxia type 1. *Nat Genet*. Jul 1993;4(3):221-6. doi:10.1038/ng0793-221

3. Sanpei K, Takano H, Igarashi S, et al. Identification of the spinocerebellar ataxia type 2 gene using a direct identification of repeat expansion and cloning technique, DIRECT. *Nat Genet*. Nov 1996;14(3):277-84. doi:10.1038/ng1196-277

4. Duyao M, Ambrose C, Myers R, et al. Trinucleotide repeat length instability and age of onset in Huntington's disease. *Nat Genet*. Aug 1993;4(4):387-92. doi:10.1038/ng0893-387

5. Laskaratos A, Breza M, Karadima G, Koutsis G. Wide range of reduced penetrance alleles in spinal and bulbar muscular atrophy: a model-based approach. *J Med Genet*. Jun 2021;58(6):385-391. doi:10.1136/jmedgenet-2020-106963

6. Mongelli A, Magri S, Salvatore E, et al. Frequency and distribution of polyQ disease intermediate-length repeat alleles in healthy Italian population. *Neurol Sci*. Jun 2020;41(6):1475-1482. doi:10.1007/s10072-019-04233-3

7. Gossye H, Engelborghs S, Van Broeckhoven C, van der Zee J. C9orf72 Frontotemporal Dementia and/or Amyotrophic Lateral Sclerosis. In: Adam MP, Everman DB, Mirzaa GM, et al, eds. *GeneReviews(®)*. University of Washington, Seattle. All rights reserved.; 1993.

8. Iacoangeli A, Al Khleifat A, Jones AR, et al. C9orf72 intermediate expansions of 24-30 repeats are associated with ALS. *Acta Neuropathol Commun*. Jul 17 2019;7(1):115. doi:10.1186/s40478-019-0724-4

9. Squitieri F, Jankovic J. Huntington's disease: how intermediate are intermediate repeat lengths? *Mov Disord*. Dec 2012;27(14):1714-7. doi:10.1002/mds.25172

10. Gardiner SL, Boogaard MW, Trompet S, et al. Prevalence of Carriers of Intermediate and Pathological Polyglutamine Disease-Associated Alleles Among Large Population-Based Cohorts. *JAMA Neurol*. Jun 1 2019;76(6):650-656. doi:10.1001/jamaneurol.2019.0423
